# Predictability of environment-dependent formation of G-quadruplex DNAs in human mitochondria

**DOI:** 10.1038/s42004-025-01532-z

**Published:** 2025-05-03

**Authors:** Lutan Liu, Shuntaro Takahashi, Sarptarshi Ghosh, Tamaki Endoh, Naoto Yoshinaga, Keiji Numata, Naoki Sugimoto

**Affiliations:** 1 FIBER (Frontier Institute for Biomolecular Engineering Research), Konan University, Chuo-ku, Kobe Japan; 2 FIRST (Graduate School of Frontiers of Innovative Research in Science and Technology), Konan University, Chuo-ku, Kobe Japan; 3 Biomacromolecule Research Team, RIKEN Center for Sustainable Resource Science, Wako-shi, Saitama Japan; 4 Institute for Advanced Biosciences, Keio University, Tsuruoka-shi, Yamagata Japan; 5 Department of Material Chemistry, Kyoto University, Kyoto-shi, Kyoto Japan

**Keywords:** DNA, Biophysical chemistry, DNA

## Abstract

Molecular crowding affects the stability of nucleic acids (DNA and RNA) and induces their non-canonical structures. As the level of molecular crowding varies spatio-temporally in cells, it would be beneficial to predict the behaviour of DNA and RNA structures depending on the local cellular environments. This has applications in human mitochondria, which possess an especially crowded condition. In this study, the predictability of guanine-quadruplex (G4) DNA formation in the environment specific to human mitochondria was investigated. In accordance with the stability of duplexes predicted by our nearest-neighbour parameters, the G-rich duplex stability was found to effectively decrease and G4 formation was induced in mitochondrion-like conditions compared to the nucleus-like conditions. Using a peptide-based mitochondrial targeting system, a G4 reporter assay performed in mitochondria indicated that G4 formation were more favoured in mitochondria more than in the nucleus. These findings provide insights useful for the prediction of the behaviour of nucleic acids in mitochondria.

## Abstract (web summary)

Molecular crowding varies spatio-temporally in cells, affecting the stability of nucleic acids and inducing their non-canonical structures. However, the exact behavior of nucleic acids in local cellular environments remains unclear. Here, the authors report guanine-quadruplex (G4) DNA formation in the environment specific to human mitochondria and demonstrate repression of gene expression by G4 formation in mitochondria.

## Introduction

Molecular crowding is a unique environment seen in intracellular loci where biological processes occur^1–4^. This condition is composed of highly concentrated cosolutes including proteins, nucleic acids, polysaccharides, various ions, and small molecules. These cosolutes alter the physicochemical properties of the solution such as the dielectric constants, water activity, and viscosity, as well as the steric effects^5^. Furthermore, molecular crowding is known to change spatiotemporally in cells. During the cell cycle, the contents and concentration of biomolecules change dynamically^6–8^. Localized cellular environments such as cell organelles, membrane surfaces, and membrane-less compartments (liquid-liquid phase separation) have different crowding environments compared to the cytoplasm^9,10^. Thus, studying the biomolecules in the statically dilute conditions in vitro is not sufficient to understand biomolecular behaviour in cells, because the molecular environments markedly affect these properties.

Among biomolecules, nucleic acids (DNA and RNA), which are negatively charged polymers, are particularly sensitive to molecular crowding^5^. Interestingly, the structural stabilities of canonical duplex and non-canonical structures such as triplexes and tetraplexes display different sensitivities to molecular crowding. For instance, duplexes are destabilized by small cosolutes^11,12^, whereas guanine-quadruplexes (G4s), which is one of the non-canonical structures, are stabilised^13–15^. Potential quadruplex-forming sequences (PQS) are widely found in the genome and are known to participate in regulatory functions in the cell^16^. Thus, the local change in molecular crowding can alter duplex-quadruplex equilibria in the cell and control gene replication and expressions. For example, mitochondria, which are key organelle for supplying the energy for the cell to exert all its functions, have its own DNAs (mtDNAs) that contain PQS^17^. G4s in mtDNAs have been suggested to play a role in the replication and transcription of mtDNAs^18–20^, which are vital for mitochondrial functions. In addition, mitochondrial G4s are associated with mtDNA deletions, leading to hereditary diseases and aging^18,21^. The analysis of the effects of mitochondrial conditions on mtDNA is undoubtedly crucial for elucidating mitochondria-specific biological processes and developing the therapeutics and related technologies for mitochondrial diseases. In particular, there is a growing interest in the molecular environments of mitochondria for its potential to induce formation of non-canonical structures on mtDNA. The content of mitochondria has been reported to be enriched with proteins^22,23^. A recent important report has suggested that macromolecular crowding in the mitochondrial matrix of human HeLa cell is much higher than that of the nucleus and cytosol^24^. These findings imply that the unique condition of mitochondria may drive G4 formation of mtDNA in a manner distinct from that in the nucleus. However, environment-dependent G4 formation in mitochondria is not well known. To clarify the roles of the non-canonical structure in mitochondria, it will be beneficial to predict the behaviour of DNA structures in mitochondrial environments.

Predicting DNA stability is important for determining the behaviour of DNA structures in a specific environment. The most widely established method for the prediction of nucleic acid duplex stability is the nearest neighbour (NN) model^25^. The NN model assumes that the total free energy change upon duplex formation can be determined from the sum of the free energy contribution of the NN base pairs^25,26^. Thus far, the NN parameters of DNA^27^, RNA^28^ and RNA/DNA hybrid^29^ duplexes have been developed. Recently, the NN parameters were characterized under various ionic^30^ and crowding^31^ conditions, allowing for the prediction of duplex stability in conditions similar to those of the intracellular environment, such as the nucleus. However, given the non-uniform nature of intracellular crowding, there is a need to establish the NN model in more diverse crowded conditions to predict nucleic acid stability more accurately in organelle-specific environments.

To develop NN parameters that are applicable to mitochondrial environments, it is necessary to first characterize the stability of mitochondrial G4 in cells. The relative stability between the duplex and G4 is a useful measure of the conformational preference of G4s over duplexes. Thus, in this study, various spectroscopic and microscopic methods were employed to investigate the propensity of mtDNA to form G4s in the mitochondria relative to the nucleus. Our findings show enhanced G4 population in mitochondrial environments compared to that of the nucleus.

## Results and Discussion

### Estimation of duplex stability in mitochondria

The stability of DNA duplexes is readily decreased with molecular crowding by polyethylene glycol with an average molecular weight of 200 (PEG200) mainly due to the decrease in water activity^5^. We have recently succeeded in the accurate prediction of duplex stabilities in various cellular environments using parameters based on the NN model for DNA duplexes, which were obtained in solutions containing different concentrations of cations and cosolutes^32,33^. In particular, we introduced “hydration parameters” for the accurate prediction of AT (adenine and thymine)- and GC (guanine and cytosine)-biased DNA duplexes, because the specific hydration patterns depending on solution environments occur on the grooves of the helices when AT and GC bases are consecutively aligned by 9 or 4 mers, respectively^33^. The prediction parameters have been designed for estimating the duplex stabilities in the solution containing 40 wt% PEG200 for mimicking the cellular condition^33^.

**Fig. 1:**
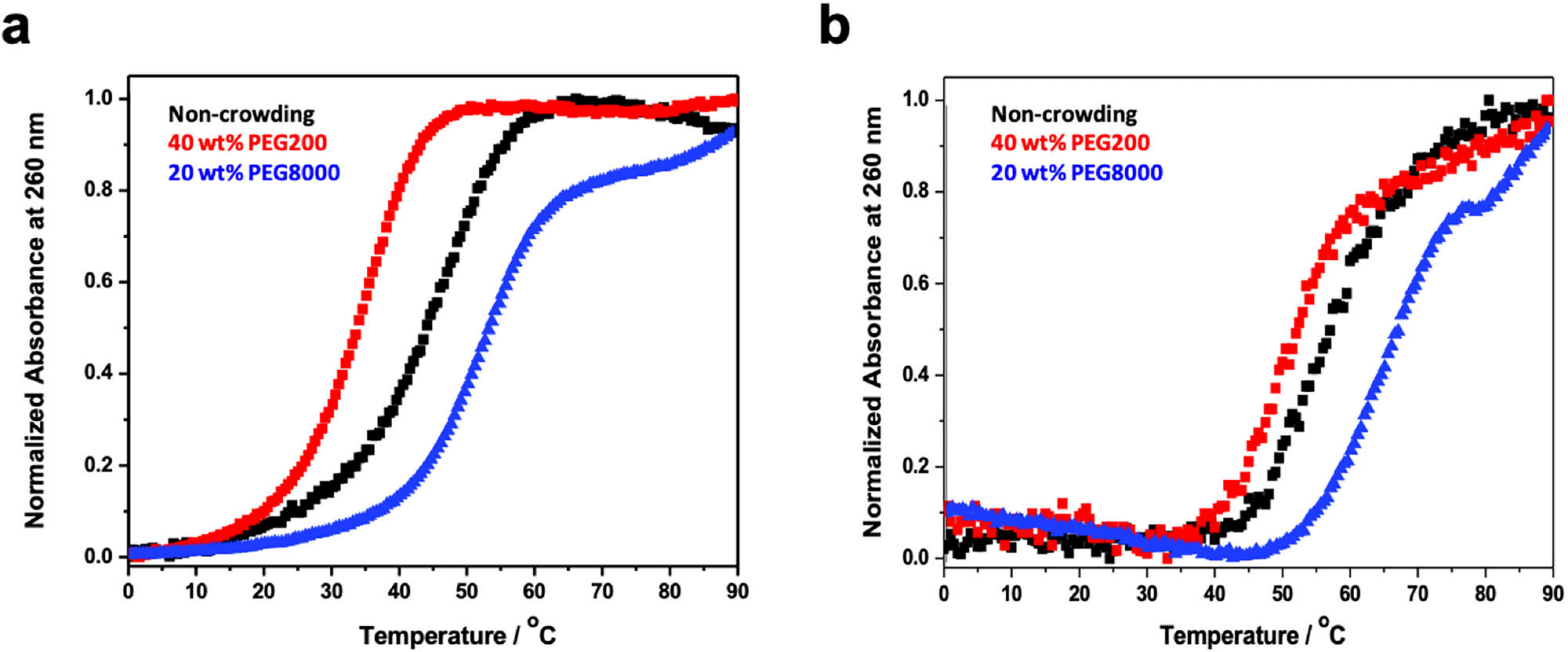
UV melting curves of unbiased and biased duplex sequences. UV melting curves of 100 µM unbiased sequence d(ATGAGCTCAT) (**a**) and GC-biased sequence d(GCGCCGC) (**b**). The melting was conductedin the non-crowding (black) or the crowding condition in presence of 40 wt% PEG200 (red) and 20 wt% PEG8000 (blue) with 10 mM Na-phosphate buffer (pH 7.0), 100 mM NaCl and 1 mM EDTA.

Since the mitochondrial matrix has been suggested to be more crowded with macromolecules compared with the cytosol and nucleus in HeLa cells^24^, we tested how the duplex formation could be predicted in the presence of large molecular weight PEG such as PEG8000. PEG8000 effectively shows a higher viscosity than PEG200, making it an ideal crowding agents for simulating the mitochondrial matrix condition^34^. We have previously reported that the destabilisation of duplexes caused by the addition of 40 wt% PEG200 is less profound in the GC-biased sequence compared to the unbiased sequence^33^. As shown in Fig. 1, the shift of melting curves is more apparent for the GC-biased sequence d(GCGCCGC) compared to the unbiased sequence d(ATGAGCTCAT) when 40 wt% PEG200 is added. ∆*G*°_37_ of d(GCGCCGC) was obtained as −9.6 kcal mol^−1^ in the non-crowding condition and −8.2 kcal mol^−1^ in the condition with 40 wt% PEG200 (Supplementary Table S1). This shows that at 37^o^C, the crowding-induced differential free energy of the biased sequence d(GCGCCGC) was smaller compared to the unbiased sequence d(ATGAGCTCAT) (+2.7 kcal mol^−1^ for d(ATGAGCTCAT) and +1.4 kcal mol^−1^ for d(GCGCCGC), respectively). The reduction of destabilisation effect by PEG200 in the GC-biased sequence is caused by the specific groove hydration along the GC-tract, as reported previously^33^.

The effect of PEG8000 on biased and unbiased sequences was also measured and compared with that of PEG200. 20 wt% PEG8000 was used to maintain a degree of crowding consistent with 40 wt% PEG200. Contrary to PEG200, PEG8000 stabilized both duplexes, since the excluded volume effect of large cosolutes facilitates the duplex formation more than the destabilisation effect from reduced water activity (Fig. 1 and Supplementary Fig. S1)^35,36^. The stabilisation effect by PEG8000 was about two-fold larger for the unbiased duplexes than that of the GC-biased duplexes (∆∆*G*°_37_ = –1.7 kcal mol^−1^ for d(ATGAGCTCAT) and ∆∆*G*°_37_ = −0.8 kcal mol^−1^ for d(GCGCCGC), respectively, shown in Supplementary Table S1). This trend indicates that PEG8000 stabilized the unbiased duplex more because the unbiased sequence d(ATGAGCTCAT) was longer than the GC biased sequence d(GCGCCGC). In contrast to PEG200, PEG8000 does not disturb the hydration network of the groove of helices^33^.

To predict the stability of duplex under crowded conditions (∆*G°*_duplex_), an NN model in molecular crowding was established as follows:

1 $$\Delta G^{\circ}_{{{\rm{duplex}}}}=\sum \Delta G^{\circ}_{{{\rm{NN}}}}+\Delta G^{\circ}_{{\mathrm{int}}}+\Delta G^{\circ}_{{{\rm{sym}}}}+\Delta G^{\circ}_{{{\rm{crowding}}}}$$

where ∆*G°*_NN_, ∆*G°*_int_ and ∆*G°*_sym_ represent the energetic contribution of the propagating base pairs, helix initiation factor, sequence symmetry factor, and molecular crowding, respectively. ∆*G°*_crowding_ is further classified as follows:

2 $$\Delta G^{\circ }_{{{\rm{crowding}}}}=\Delta G^{\circ }_{{{\rm{water}}}\,{{\rm{activity}}}}+\Delta G^{\circ }_{{{\rm{exc}}}.\, {{\rm{vol}}}.}+\Delta G^{\circ }_{{{\rm{hydration}}}}$$

where ∆*G°*_NN_, _water activity_, ∆*G°*_exc. vol._ and ∆*G°*_hydration_ represent the energetic contributions of water activity, excluded volume effect, and hydration obtained from the hydration parameter^33^. All of the free energy terms can be calculated using the parameters established in our previous reports^32,33^. Thus, the contribution of each free energy terms to the stabilities of unbiased and biased sequences in the presence of either PEG200 or PEG8000 was predicted. To reduce the effects from excluded volume depending on the length of DNA strands, we selected the same 10-mer sequences (Table 1). For the sake of comparison, similar concentrations (20 wt%) of PEG200 and PEG8000 were used. In the 20 wt% PEG200 condition, the predicted ∆*G*°_37 crowding_ of the unbiased 10-mer d(GGCAGCTGCC) was 1.73 kcal mol^−1^, whereas that of the GC-biased 10-mer d(GCGGCGCCGC) was 1.02 kcal mol^−1^. The difference between the two predicted values (∆∆*G*°_37 crowding_ = ∆*G*°_37 crowding_ (GC-biased) – ∆*G*°_37 crowding_ (unbiased)) was −0.71 kcal mol^−1^. The additional ∆*G*°_37 hydration_ of the GC-biased duplex (−1.35 kcal mol^−1^) effectively cancel the energetical cost of the water activity of the GC-biased sequence (∆*G*°_37 water activity_), indicating that ∆∆*G*°_37 crowding_ is mainly derived from ∆*G*°_37 hydration_. Thus, the stabilizing effect of molecular crowding by PEG200 was much higher for the GC-biased sequence than for the unbiased sequence. In contrast,in the PEG8000 condition resembling the mitochondrial matrix, ∆∆*G*°_37 crowding_ was +0.38 kcal mol^−1^, indicating that PEG8000 can destabilize GC-biased DNA duplexes more than PEG200 due to the hydration effect. As G4s are formed from the GC-biased sequences, the destabilisation of GC-biased duplexes in the PEG8000 condition suggests that G4 formation is facilitated in the mitochondrial matrix compared to the nucleus, in which the crowding condition is modified by PEG200^31^.

**Table 1:** Predicted thermodynamic parameters for ∆*G*°_37 crowding_ DNA duplex formation^a^

Sequences^a^	Cosolute	∆*G˚*_37 water activity_^b^ (kcal mol^−1^)	∆*G˚*_37 exc. vol._^c^ (kcal mol^−1^)	*∆G˚*_37 hydration_^b^ (kcal mol^−1^)	∆*G˚*_37 crowding_ (kcal mol^−1^)
Unbiased	20 wt% PEG200	2.77	−1.04	0	1.73
GC-biased	20 wt% PEG200	3.41	−1.04	−1.35	1.02
Unbiased	20 wt% PEG8000	2.21	−1.17	0	1.04
GC-biased	20 wt% PEG8000	2.59	−1.17	0	1.42
Unbiased	40 wt% PEG200	5.54	−2.08	0	3.46
GC-biased	40 wt% PEG200	6.81	−2.08	−2.71	2.02

^a^Calculations were in the condition containing 20% PEG200 and 20 wt% PEG8000 in 10 mM phosphate buffer (pH 7.0) with 100 mM NaCl and 1 mM EDTA.

The unbiased d(GGCAGCTGCC) (10 mer) and GC-biased d(GCGGCGCCGC) (10 mer) were used with each complementary DNA strand.

^b^Values were predicted using parameters previously reported by our group^32,33^.

^c^Values were predicted using the parameters corrected for 100 mM NaCl using the method of Huguet et al. ^48^.

In addition to the effect of cosolute size, cosolute concentration also influences the stability of DNA by altering the viscosity of the solution. The energetic costs of molecular crowding in 20 wt% and 40 wt% PEG200 are shown in Table 1. For unbiased sequence, the ∆∆*G*°_37 crowding_ in 40 wt% PEG200 was −1.46 kcal mol^−1^, which was almost two-fold of that in 20 wt% PEG200. The ∆*G*°_37 crowding_ of the GC-biased duplex in 40 wt% PEG200 was also nearly twice of that in the case of 20 wt% PEG200. Considering that the G4 formation is accelerated by the decrease in water activity of the solution^13–15^, a concentrated cosolute would also facilitate the G4 formation in the mitochondrial matrix by the simultaneous effect of duplex destabilisation and G4 stabilisation.

### Preferential G4 formation in the mitochondria-mimicking condition

It has been previously demonstrated that the G4 structure is markedly stabilised in the presence of a cosolute with large molecular weight, such as PEG8000, compared with a small cosolute^37^ and cosolute concentrations that induce decrease in water activity^13–15^, suggesting the preferential formation of G4 structures in the GC-biased regions in mtDNAs. To further confirm that G4 conformation is preferred under potential mitochondria conditions, we performed CD spectroscopy of the duplexes that contain G4-forming sequences to deduce the level of G4 formation under nucleus- and mitochondria-like conditions. Our previous finding showed that the addition of 30 wt% 1,3-propanediol (PDO) as a cosolute with 100 mM NaCl well-reproduced the RNA duplex stability directly measured in the nucleus^31^. Thus, this condition was chosen as the reference condition representing the nucleus-like environment. 1,3-PDO was expected to exert a similar effect as PEG200 on biased duplex and G4, since ethylene glycol, a small cosolute like 1,3-PDO, was found to have similar effects on hydration-induced stabilisation in biased DNA as PEG200^33^. The mitochondria conditions were simulated according to the viscosity difference between the nucleus and mitochondria matrix, which are estimated to be approximately 1.1 cP~1.4 cP^38,39^ and 3.69 cP~4.57 cP^24^ at 37 °C, respectively. The viscosities of the buffers were adjusted to match that of mitochondria by mixing additional cosolutes, such as 1,3-PDO up to 60 wt% or PEG8000 up to 1 wt%, to the reference condition. The types of cosolutes were chosen to address the crowding effects of both small cosolute and large macromolecules, which exhibit distinct crowding effects^5^. Although reduction in water activity by the crowders can drive the formation of A-DNA in GC-rich sequences, previous studies by our group have shown that the level of crowding similar to the ones applied in this study does not cause conformational switching of the duplexes to A-DNA^32,33^. The viscosities of each solution are listed in Supplementary Table S2. To mimic the cellular ionic environment, 150 mM KCl was used instead of NaCl. The DNA sequences were designed from our previous study of in-cell transcription assay regulated by G4 formation derived from the *cMyc* gene^40^ and PQSs derived from human mitochondria^41^ (Table 2 and Supplementary Table S3).

**Table 2:** G4-forming DNA duplex sequences used in the NMM assay, CD spectroscopy and the cell-based assays

Name^a^	Sequence (5’ – 3’)^b^
D1^c^	GGGAGCCAGGGACGGCCGGG
D2^c^	GGGGACAGGGGCGGGGTGGG
D4^c^	GGGGGGATCAGCGGGAGGGCTGGG
mt6363	GGGACGCGGGCGGGGGGATATAGGG
CSBII	GCGGGGGAGGGGGGGTTTG
L^c^	TTGGGTTGTAACTATCGAGG
KSS^d^	GGGGAGGGGTGTTTAAGGGGTGGCTAGGG
PMPS^d^	GGGACGCGGGCGGGGATATAGGG
HRCC^d^	GGGGGTTGGGTATGGGGAGGGGGG

^a^All oligonucleotides used in this study were shown in the Supplementary Table 2.

^b^Complementary sequences are not shown.

^c^The sequences are from our previous study^40^.

^d^The sequences are derived from mitochondrial gene^41^.

The extent of G4 formation under mitochondrial-like conditions were first examined with the assay using N-methyl mesoporphyrin IX (NMM) (Supplementary Fig. 2a). NMM is a compound that exhibit high selectivity for G4 over duplexes that exert strong fluorescence signal upon binding to G4s^42^. The G4-forming sequences in the presence of 150 mM KCl showed a clear NMM fluorescence peak at 610 nm, indicating G4 formation by the sequences^43^. As expected, no profound change in the signal strength was observed for non-G4 forming L and D1 series. The relative change in the fluorescence intensity of the sequences at 610 nm is shown in Supplementary Fig. 2b. The fluorescence intensity increased with the addition of cosolutes for all sequences. These results suggest that G4 formation in mitochondria are accelerated by the mitochondrial condition.

**Fig. 2:**
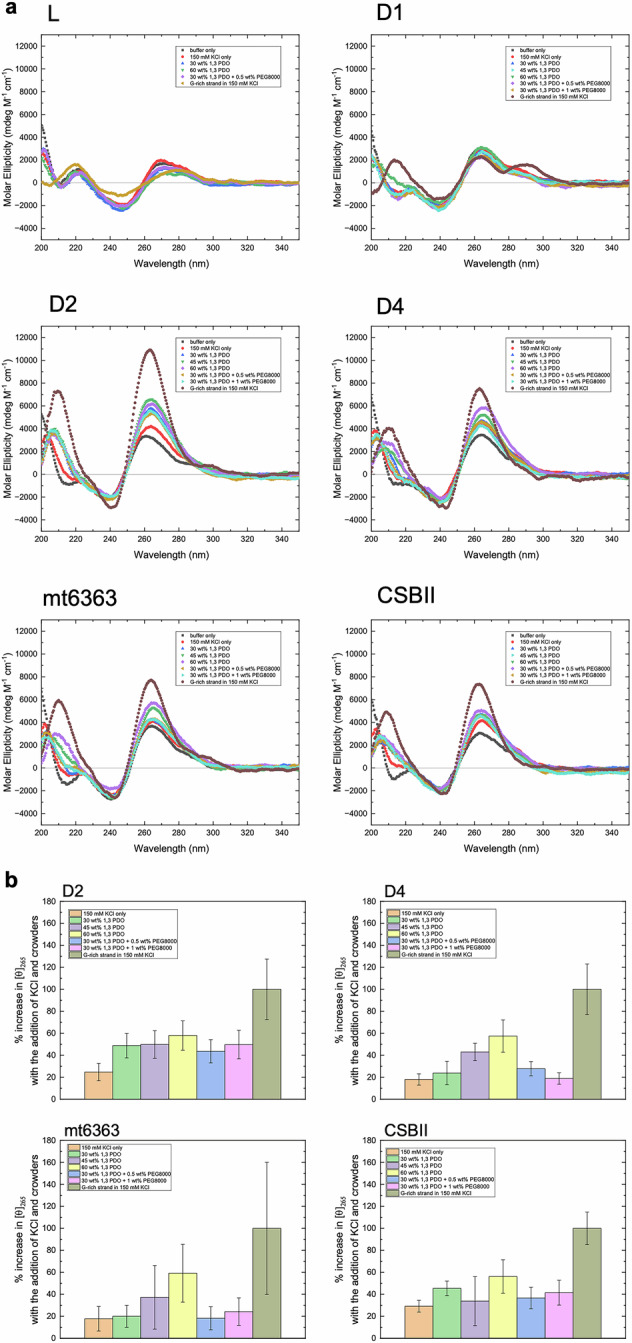
CD analysis of G4 formation by double-stranded DNA in mitochodnria-mimicking conditions. **a** CD spectra of the double-stranded sequences of L, D1, D2, D4, mt6363, and CSBII. The spectra were measured in 10 mM Na_2_HPO_4_ and 1 mM EDTA buffer (pH 7.0) with the following additions: (i) buffer only, (ii) 150 mM KCl, (iii) 150 mM KCl and 30 wt% 1,3-PDO, (iv) 150 mM KCl and 45% 1,3-PDO, (v) 150 mM KCl and 60 wt% 1,3-PDO, (vi) 150 mM KCl, 30 wt% 1,3-PDO, and 0.5 wt% PEG8000, and (vii) 150 mM KCl, 30 wt% 1,3-PDO, and 1 wt% PEG8000. The CD spectra of the L-s, D1-s, D2-s, D4-s, mt6363-s, and CSBII-s strands in the buffer and 150 mM KCl without crowders were also included. **b** The relative increase in the molar ellipticity at 265 nm of the double-stranded D2, D4, mt6363, and CSBII sequences. The signals of duplexes in the absence of KCl and cosolutes are defined as 0%, while the signals of single-stranded G-rich strands in the presence of 150 mM KCl are defined as 100%. The experiment was conducted in triplicate, and the standard deviations are represented by the error bars.

Next, to investigate the effect of solution environment on G4 formation more precisely, circular dichroism (CD) spectroscopy was applied on select sequences. Fig. 2a shows CD spectra of series D1, D2, D4, mt6353 and CSBII measured at 37 ^o^C. We also tested the non-G4 forming sequence, denoted as L, as a control. For each series, the single-stranded -s sequence (e.g., D1-s) or duplexes of -s/-as sequences (e.g., a duplex from D1-s/D1-as) were used. For the single-stranded -s sequence, in the presence of 150 mM KCl, D2-s, D4-s, mt6363-s and CSBII-s (denoted as “G-rich strand in 150 mM KCl” in the inset of Fig. 2a showed clear signs of parallel topology G4 formation, as indicated by the positive peaks at 210 and 265 nm^44^. On the other hand, D1-s showed a mixed-type G4 topology, with positive peaks at 265 and 295 nm as previously reported^40^. L-s did not show any signatures of G4 due to the absence of long G-tracts required for stable G4 formation. For the duplexes, in the absence of cosolutes and KCl, all sequences exhibited similar CD spectra with low peak levels, indicating that the G4 did not form without K^+^ ions. In the presence of 150 mM KCl without the crowders, the duplexes containing G-rich strands showed elevated peak levels. As cosolutes were added, the increase in peak height was observed for all duplexes with G4 forming sequences, suggesting an increase in G4 formation. The relative change in the molar ellipticity at 265 nm, [*ϴ*]_265_, of D2, D4, mt6363 and CSBII sequences is shown in Fig. 2b. Herein, 0% intensity is defined as the molar ellipticity of each duplex sequence in the absence of 150 mM KCl and crowders, representing no formation of G4 by the G-rich strand. 100% intensity is defined as the molar ellipticity of single-stranded G-rich strands, representing the intensity at which all G-rich strands form G4. The % change in [*ϴ*]_265_ of the DNA in each condition (%[*ϴ*]_265_), which represents the level of G4 formation by the G-rich strand, was computed as $100* \frac{X-B}{G-B},$ where X is the [*ϴ*]_265_ of the DNA, B is the [*ϴ*]_265_ of DNA in the absence of KCl and crowders, and G is the [*ϴ*]_265_ of single-stranded G-rich strand in the presence of 150 mM KCl. For all four sequences, the signal strength increased with the addition of the cosolutes. D2 and CSBII showed a near two-fold formation of G4 compared to mt6363, especially in the presence of PEG8000, indicating that the sequence with longer G-tracts (i.e. higher GC biased sequences) were more sensitive to the presence of cosolutes, inducing G4 formation. The relative ratio of %[*ϴ*]_265_ in the mitochondria-like conditions with respect to the nucleus-like conditions (i.e., $\frac{ \% {[\theta ]}_{265}}{ \% {{[\theta ]}_{265}}_{30{{\rm{wt}}} \% {{\rm{PDO}}}}}$) are shown in Table 3. Compared to the solution condition resembling the nucleus condition including 30 wt% 1,3-PDO, the level of G4 formation varied with the additive cosolutes (Table 3). For D2 duplex, the G4 formation was promoted by higher concentrations of 1,3-PDO, whereas the addition of PEG8000 did not effectively increase the G4 formation. In fact, only a small increase was observed for D4 with additional 0.5% PEG8000 but not for others. These results could be related to the length of GC tract within the sequence, since the total number of GC base pairs in the tract was 15 for D4 and maximum 13 for others.

**Table 3:** Relative Increase in G4 formation in mitochondria-like conditions with respect to nucleus-like condition

Solution	D2	D4	mt6363	CSBII
30 wt% 1,3-PDO (nucleus-like condition)	1	1	1	1
45 wt% 1,3-PDO	1.02	1.80	1.86	0.74
60 wt% 1,3-PDO	1.19	2.41	2.95	1.24
30 wt% 1,3-PDO + 0.5 wt% PEG8000	0.89	1.17	0.91	0.81
30 wt% 1,3-PDO + 1 wt% PEG8000	1.02	0.79	1.21	0.91

To ensure that there is no significant amount of excess G-rich ssDNA in the dsDNA samples used in the CD spectroscopy and NMM assay, we performed native PAGE analysis of the dsDNA samples used in these experiments (Supplementary Fig. S3). The band intensity of the single-stranded region within the dsDNA lanes were compared with either the intensity of the dsDNA band in the same lane or the G-rich ssDNA band in the ssDNA lane. The % intensity of the ssDNA region is listed in Supplementary Table S4. Overall, most sequences only showed a single band at the 20bp dsDNA region in the dsDNA lanes. The band intensities at the ssDNA regions were less than 5% of those of the dsDNA and G-rich ssDNA bands, indicating that there is insignificant amount of excess G-rich strand within the duplex samples. To further assure that the probable presence of excess G-rich strand has minimal effect on our NMM assay and CD spectroscopic studies, we additionally performed similar CD spectroscopic analysis of D2, D4, mt6363, and CSBII dsDNA in the presence of 20% excess C-rich strand relative to the complementary G-rich strand (Supplementary Fig. S4 and Supplementary Table S5). We observed that the general trend in crowding-induced enhancement of G4 formation remained similar regardless of the presence of excess C-rich strands. As well, the relative increase in G4 population with respect to the nucleus condition also remained similar. Thus, the presence of excess G-rich strand does not significantly affect the outcome of our assays.

Overall, our findings indicate that the G4 formation in mitochondria may be promoted in a sequence-dependent fashion compared to that in the nucleus if the mitochondrial condition can be approximated in a PEG8000-based solution. On the other hand, as observed in the preferential formation of G4 in the solution containing 45 and 60 wt% 1,3-PDO, the mitochondrial condition may exert a large decrease in water activity via dense cosolutes, inducing a marked destabilisation in the duplex and promotion of G4s.

### Repression of gene expression by G4 formation in mitochondria

**Fig. 3:**
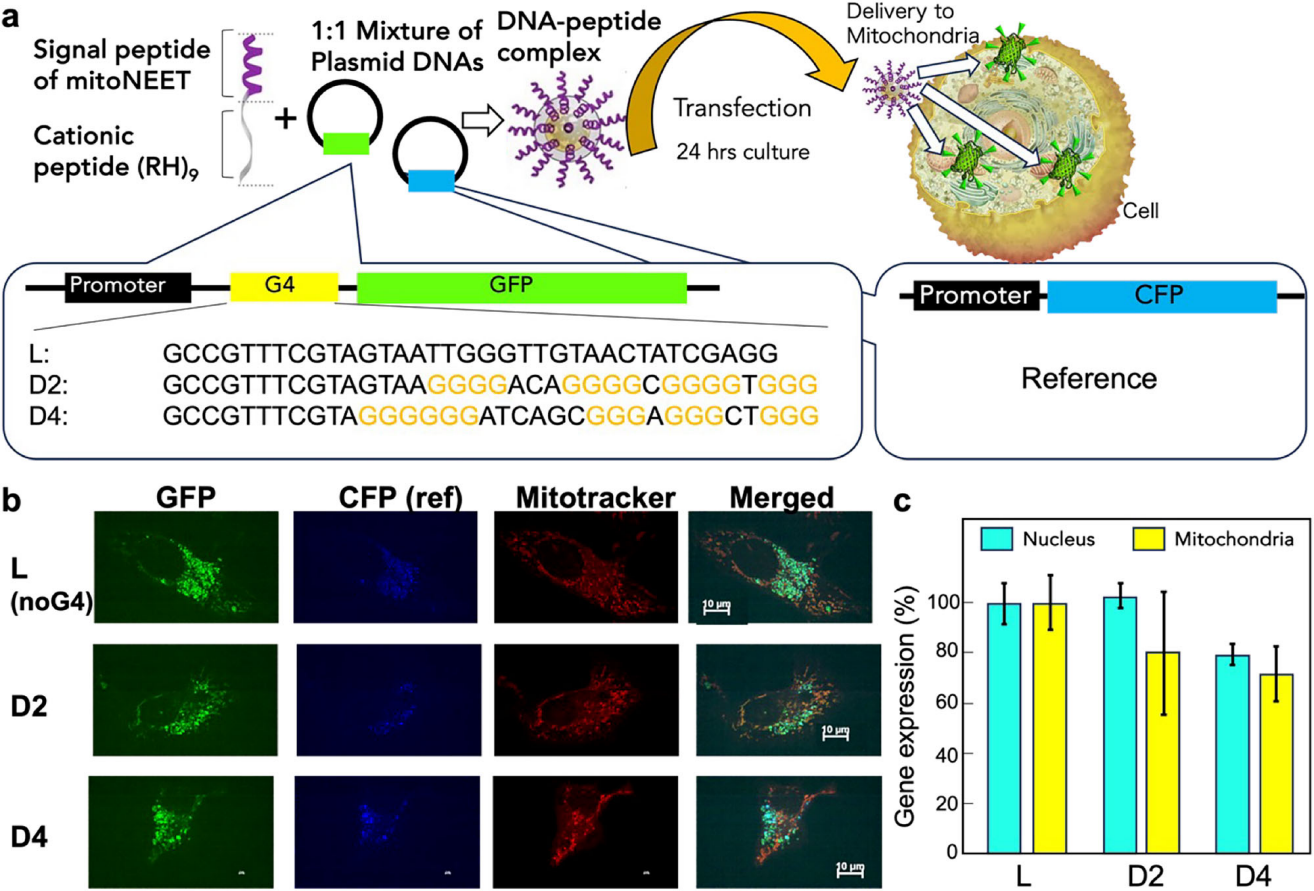
G4 formation in intracellular mitochondria. **a** Schematic illustration of mitochondrial targeting gene expression regulated by G4 forming sequence. The images were partially adapted from Chuah et al. ^49^ with permission from ACS. **b** Images of confocal microscopy of HeLa cells transfected with L, D2, and D4 sequences containing plasmid DNAs. **c** Relative expression level of GFP of HeLa cells. The signals were normalized with those from the cells infected with L-plasmid DNAs as 100%. The expression levels of nucleus were taken in the previous study^40^. GFP expression in mitochondria by D4 sequences was significantly lower than that by the control sequence, as indicated by two-tailed t-test (*p* = 0.0352, *n* = 3 independent experiments). The effect size of the GFP expression in mitochondria and nucleus, measured by Cohen’s d, was 1.35 and 0.80 for D2 and D4 sequences, respectively, indicating that the effect size is large. Thus, we conclude that there is a practical significance between the measurements in nucleus and mitochondria for both sequences.

To investigate the effect of the G4 formation on gene expression by mtDNAs, a previously established peptide carrier was utilized^45,46^ to derive reporter vectors into mitochondria (Fig. 3a). A series of plasmid DNA vectors were constructed consisting of a promoter sequence that was only recognized by the mitochondrial RNA polymerase and PQSs, which were used in the CD and NMM assays, in between the promoter and the open reading frame of green fluorescent protein (GFP) (Fig. 3a). The PQS have been demonstrated previously to repress the transcription of reporter genes in human cancer cell lines because the formation of G4 on the transcriptional template hinders the progression of human RNA polymerase II along the DNA^40^. For use as a control vector, a vector containing the cyan fluorescent protein (CFP) gene without PQS was constructed.

The co-transfection of the reporter vector and the control vector and the evaluation of the fluorescence intensity of GFP relative to that of CFP in the mitochondria facilitated the analysis of the effect of G4 formation on the transcription repression in mitochondria. The peptide carrier was mixed with the reporter and the control vectors, and the complex was added to HeLa cells to perform transfection into mitochondria. After incubation for 24h, the fluorescence of reporter proteins was imaged by confocal microscopy (Fig. 3b). The granular fluorescence of GFP as well as CFP was observed in cells treated with the reporter vector containing linear (L) sequence, as observed in previous studies^40^. Almost all the fluorescent granules merged with the signals of Mitotracker, which is a fluorescent probe for mitochondria, suggesting that the GFP and CFP were expressed in the mitochondria. Similarly, the cells transfected with the reporter vector containing D2 and D4 sequences expressed GFP and CFP without a G4 formation sequence before the coding region. The merged fluorescence imaged in pseudo colour showed cyanic granules in cells transfected with the vector containing D4 sequence, indicating that the expression of GFP was reduced relative to the CFP expression. Next, the fluorescence intensities of GFP relative to CFP (GFP/CFP) were quantified in each cell. The gene expression levels of the reporter were evaluated from the GFP/CFP values after normalisation using the GFP/CFP value calculated from cells transfected with the reporter vector containing the L sequence. Interestingly, D2 and D4 showed lower levels of GFP expression. In the previous report, D2 did not suppress the gene expression compared to the expression from L sequence in nucleus of MCF-7 cells^40^. The results indicated that the repression of gene expression is more effective in mitochondria compared to the nucleus.

Based on the in vitro behaviours of duplex and quadruplexes, which suggest preferential G4 formation in mitochondria-like macromolecular crowding, the GC-biased regions in mtDNA may impact the mitochondrial gene expressions through the formation of G4 structures. More practically, 45 or 60 wt% 1,3-PDO-containing solutions promoted D2 G4 formation, suggesting that the mitochondrial conditions can be approximated by the solutions with a larger decrease in the level of water activity by keeping a high viscosity compared with the nucleus. Since each mitochondrion is not uniform due to heteroplasmy, the relatively large error level of D2 may indicate the possibility of different statuses of the mitochondria. This is likely to be the topic of future work. These findings provide insights with which to predict the G4 formation on mtDNA and their biological significance by enabling the prediction of the stability of both duplexes and quadruplexes in various mitochondrial conditions. These predictions provide critical information regarding G4-mediated mitochondrial functions depending on the different cellular environments.

## Conclusion

In summary, we demonstrated that G4s can form preferentially in mitochondria compared to the nucleus. Although data of the stability of DNA duplexes is lacking, information on the viscosity of the mitochondria matrix and the nucleus provided the potential conditions for approximating the mitochondrial matrix. Our in vitro and cell-based assays indicated that the environment of the mitochondrial matrix provided preferential formation for the DNA of G4s. The mitochondrial condition was markedly altered by the conditions external to the cells. Moreover, the molecular properties of the mitochondria were relatively unique, such as ATP concentrations, compared with the nucleus. Taken together, these results provide information to support the accurate prediction of the stability of DNA duplexes in mitochondria, as well as insights into the establishment of simulations.

## Methods

### Materials

High-performance liquid chromatography-purified DNA oligonucleotides were purchased from Japan Bio Services Co., Ltd. DNA primers were purchased from Eurofins Genomics. The DNA samples were dissolved in Milli-Q water and stored at −20 °C until use. The concentrations of the oligonucleotides were determined by measuring the average absorbance from 90 to 80 °C at 260 nm using the extinction coefficients^47^. PEG200, PEG8000, and other reagents for buffer solutions purchased from Wako Pure Chemical Industries were used without further purification. pH of the buffer was adjusted to 7.0 at 25 °C after adding the cosolute to maintain the pH of the solution.

### UV melting study

The absorption spectra were recorded on a Shimadzu 1800 spectrophotometer with a thermoprogrammer. For the melting experiments, 10–12 concentrations of freshly prepared oligonucleotides were varied over a 50–100-fold range. The DNAs were dissolved in 10 mM phosphate buffer with 1 mM EDTA and 100 mM NaCl at pH 7.0. For the solution including a cosolute, 20 wt% PEG200 or PEG8000 was added in the buffer solution above. The sample solutions were incubated at 90 °C for 5 min, followed by a decrease in temperature to 0 °C at a rate of 1 °C min^−1^ to anneal the duplexes. Samples were stored at 0 °C for 5 min. Thereafter, the samples were heated to 90 °C at a rate of 0.5 °C min^−1^ to melt the duplexes. Water condensation on the exterior of the cuvette at low temperatures was prevented by flushing it with a constant stream of dry N_2_ gas.

### Thermodynamic analysis

The thermodynamic parameters for DNA duplex formation were determined from *T*_m_^−1^ vs. ln (*C*_t_/*s*) plots, as described in our earlier studies^32,36^. From the slope and intercept of the linear plots, the thermodynamic parameters were calculated using the following equations:

3 $${T}_{{{\rm{m}}}}^{-1}=R \, {{\mathrm{ln}}}\, ({C}_{{{\rm{t}}}}/s)/\Delta H^{\circ} +\Delta S^{\circ} /\Delta H^{\circ}$$

4 $${\Delta G^{\circ} }_{37}=\Delta H^{\circ} -310.15 \cdot \Delta S^{\circ}$$

where *T*_m_ is the melting temperature, *R* is the gas constant, *C*_t_ is the total strand concentration of the oligonucleotides; and *s* reflects sequence symmetry, which is 1 and 4 for self-complementary and non-self-complementary sequences, respectively. Herein, ∆*H*°, ∆*S*°, and ∆*G*°_37_ represent the changes in enthalpy, entropy, and free energy at 37 °C for the duplex formation, respectively. Following the standard practice for the calculation of the thermodynamic parameters, the difference between the heat capacities (Δ*C*_p_) of the single-strands and duplexes was assumed to be zero^28^.

### Native PAGE

dsDNA and ssDNA samples were analysed in 15% polyacrylamide gel at 150 V and 4 °C for 2.5 h in TBE buffer. Prior to loading, the DNA samples were annealed at 1 °C min^−1^ from 95 °C to 25 °C in in 10 mM Li_2_HPO_4_, 1 mM Li_2_EDTA, and 100 mM LiCl at pH 7.0. The gel images were captured using a Fluoreimager FLA-5100 (Fujifilm) after staining with SYBR Gold (Thermo Fisher Scientific). Peak profiles and band intensities were obtained using Fujifilm Multi Gauge software Ver 2.2.

### NMM assay for transition from the duplexes to G4s

1.5 μM dsDNA or ssDNA samples were prepared in 10 mM Na_2_HPO_4_ and 1mM EDTA buffer (pH 7.0) with the following additions: (i) buffer only, (ii) 150 mM KCl, (iii) 150 mM KCl and 30 wt% 1,3-PDO, (iv) 150 mM KCl and 45 wt% 1,3-PDO,(v) 150 mM KCl and 60 wt% 1,3-PDO, (vi) 150 mM KCl, 30 wt% 1,3-PDO and 0.5 wt% PEG8000, and (vii) 150 mM KCl, 30 wt% 1,3-PDO and 1 wt% PEG8000. The samples were annealed at 1 ^o^C min^−1^ from 95 ^o^C to 25 ^o^C. After annealing, each sample was mixed with 6 μM NMM. The emission measurements were taken at 37 ^o^C with a BioTek Cytation 5 Cell Imaging Multimode Reader with the excitation wavelength of 440 nm.

### CD spectroscopy

dsDNA or ssDNA samples (10 μM) were prepared in 10 mM Na_2_HPO_4_ and 1 mM EDTA buffer (pH 7.0) with the following additions: (i) buffer only, (ii) 150 mM KCl, (iii) 150 mM KCl and 30 wt% 1,3-PDO, (iv) 150 mM KCl and 45 wt% 1,3-PDO,(v) 150 mM KCl and 60 wt% 1,3-PDO, (vi) 150 mM KCl,30 wt% 1,3-PDO and 0.5 wt% PEG8000, and (vii) 150 mM KCl, 30 wt% 1,3-PDO and 1 wt% PEG8000. The samples were annealed at 1 °C min^−1^ from 95 °C to 25 °C before measurement. After annealing, the CD spectra of each sample were obtained on a JASCO J-1500 spectropolarimeter equipped with a temperature controller. The CD spectra were measured from 200 to 350 nm in 1.0 mm pathlength cuvettes with a scan rate of 100 nm min^−1^. The experimental temperature was 37 °C. The average of three scans were taken as the final spectra of the samples.

### G4 dependent transcription assay in mitochondria

A plasmid encoding GFP with the cox2 promoter (pDONR-cox2:gfp)^45^ was used to construct the plasmids for GFP reporter assay. To construct the plasmid DNAs, we separately amplified the DNA fragments containing the promoter region, GFP region, and the rest of the plasmid using DNA primers (Supplementary Table S6) by PCR. These three fragments were connected by using In-Fusion HD Cloning Kit (Clontech) to obtain plasmid DNA containing the HindIII and NdeI restriction sites immediately before the GFP start codon. After digestion of this plasmid DNA with HindIII and NdeI, the phosphorylated DNA adaptors (L, D1 and D4 annealed from -s and -as sequences in Supplementary Table S3) containing each G4 sequence were ligated to obtain the expression plasmid DNAs. We also replaced the GFP gene with CFP from the CFP-encoding plasmid using In-Fusion HD Cloning Kit (Clontech). To simultaneously express CFP and GFP simultaneously in the mitochondria, we mixed the obtained GFP plasmid containing each G4 forming sequence with the CFP plasmid without G4 forming sequence at a 1:1 ratio. The two plasmids was mixed with the peptide mitoNEET-(RH)_9_ at the ratio described in previous study^45^. HeLa cells were transfected with the plasmid and the peptide complex according to a previous study^45^. After 24 hours culture, the fluorescence signals of CFP and GFP were imaged using a spinning disc confocal microscopy system (CSU-W1, Yokogawa) using 405 nm and 488 nm excitation lasers and 447 nm and 525 nm emission filters for the imaging of CFP and GFP, respectively. The cells were treated with MitoTracker Red CMXRos to analyse the co-localisation of CFP and GFP signals with mitochondria^45^. The fluorescence signals of MitoTracker Red CMXRos were imaged using 561 nm excitation laser and a 600 nm emission filter using CSU-W1. The fluorescence intensity of GFP relative to that of CFP (GFP/CFP) was calculated using NIS-Elements software (NiKon). The effect of G4 forming sequence on GFP expression was analysed by normalising the GFP/CFP intensities to those obtained from the L sequence (non-G4 forming sequence) as 100%. To compare the results obtained in the nucleus previously^40^, the relative expression level were reevaluated.

### Reporting summary

Further information on research design is available in the Nature Portfolio Reporting Summary linked to this article.

## Supplementary information

Supplementary information

Description of Additional Supplementary Files

Supplementary Data 1

Supplementary Data 2

Supplementary Data 3

Supplementary Data 4

Reporting summary

## Data Availability

The corresponding data of this study is available within the paper and the Supplementary Information. The source data is available in Supplementary Data (Supplementary Data 1: Source data - CD spectra, Supplementary Data 2: Source data - GFP reporter assay, Supplementary Data 3: Source data - Native PAGE and Supplementary Data 4: Source data - NMM assay).

## References

[1] Ellis, R. J. & Minton, A. P. Join the crowd. *Nature* **425**, 27–28 (2003). 12955122 10.1038/425027a

[2] Ellis, R. J. Macromolecular crowding: an important but neglected aspect of the intracellular environment. *Curr. Opin. Struct. Biol.* **11**, 114–119 (2001). 11179900 10.1016/s0959-440x(00)00172-x

[3] Kuznetsova, I., Turoverov, K. & Uversky, V. What macromolecular crowding can do to a protein. *Int. J. Mol. Sci.* **15**, 23090–23140 (2014). 25514413 10.3390/ijms151223090 PMC4284756

[4] Subramanya, A. R. & Boyd-Shiwarski, C. R. Molecular crowding: Physiologic sensing and controll. *Annu. Rev. Physiol.* **86**, 429–452 (2024). 37931170 10.1146/annurev-physiol-042222-025920 PMC11472293

[5] Nakano, S.-I., Miyoshi, D. & Sugimoto, N. Effects of molecular crowding on the structures, interactions, and functions of nucleic acids. *Chem. Rev.* **114**, 2733–2758 (2014). 24364729 10.1021/cr400113m

[6] Shibata, D., Kajimoto, S. & Nakabayashi, T. Label-free tracking of intracellular molecular crowding with cell-cycle progression using Raman microscopy. *Chem. Phys. Lett.* **779**, 138843 (2021).

[7] Cookson, N. A., Cookson, S. W., Tsimring, L. S. & Hasty, J. Cell cycle-dependent variations in protein concentration. *Nucleic Acids Res.* **38**, 2676–2681 (2010). 20019065 10.1093/nar/gkp1069 PMC2860113

[8] Lecinski, S. et al. in *Current Topics in Membranes* 88 (eds Michael A. Model & Irena Levitan) 75-118 (Academic Press, 2021). 10.1016/S1063-5823(21)00040-5 34862034

[9] Sasmal, D. K., Ghosh, S., Das, A. K. & Bhattacharyya, K. Solvation dynamics of biological water in a single live cell under a confocal microscope. *Langmuir* **29**, 2289–2298 (2013). 23336846 10.1021/la3043473

[10] Shin, Y. & Brangwynne, C. P. Liquid phase condensation in cell physiology and disease. *Science* **357**, eaaf4382 (2017). 28935776 10.1126/science.aaf4382

[11] Nakano, S.-I., Yamaguchi, D., Tateishi-Karimata, H., Miyoshi, D. & Sugimoto, N. Hydration changes upon DNA folding studied by osmotic stress experiments. *Biophys. J.* **102**, 2808–2817 (2012). 22735531 10.1016/j.bpj.2012.05.019 PMC3379618

[12] Spink, C. H. & Chaires, J. B. Effects of hydration, ion release, and excluded volume on the melting of triplex and duplex DNA. *Biochemistry* **38**, 496–508 (1999). 9890933 10.1021/bi9820154

[13] Xue, Y. et al. Human Telomeric DNA Forms Parallel-Stranded Intramolecular G-Quadruplex in K^+^ Solution under Molecular Crowding Condition. *J. Am. Chem. Soc.* **129**, 11185–11191 (2007). 17705383 10.1021/ja0730462

[14] Miyoshi, D., Karimata, H. & Sugimoto, N. Hydration regulates thermodynamics of G-quadruplex formation under molecular crowding conditions. *J. Am. Chem. Soc.* **128**, 7957–7963 (2006). 16771510 10.1021/ja061267m

[15] Arora, A. & Maiti, S. Stability and Molecular Recognition of Quadruplexes with Different Loop Length in the Absence and Presence of Molecular Crowding Agents. *J. Phys. Chem. B* **113**, 8784–8792 (2009). 19480441 10.1021/jp809486g

[16] Varshney, D., Spiegel, J., Zyner, K., Tannahill, D. & Balasubramanian, S. The regulation and functions of DNA and RNA G-quadruplexes. *Nat. Rev. Mol. Cell Biol.* **21**, 459–474 (2020). 32313204 10.1038/s41580-020-0236-x PMC7115845

[17] Falabella, M., Fernandez, R. J., Johnson, F. B. & Kaufman, B. A. Potential roles for G-Quadruplexes in mitochondria. *Curr. Med. Chem.* **26**, 2918–2932 (2019). 29493440 10.2174/0929867325666180228165527 PMC6113130

[18] Doimo, M. et al. Enhanced mitochondrial G-quadruplex formation impedes replication fork progression leading to mtDNA loss in human cells. *Nucleic Acids Res.* **51**, 7392–7408 (2023). 37351621 10.1093/nar/gkad535 PMC10415151

[19] Agaronyan, K., Morozov, Y. I., Anikin, M. & Temiakov, D. Replication-transcription switch in human mitochondria. *Science* **347**, 548–551 (2015). 25635099 10.1126/science.aaa0986 PMC4677687

[20] Wanrooij, P. H., Uhler, J. P., Simonsson, T., Falkenberg, M. & Gustafsson, C. M. G-quadruplex structures in RNA stimulate mitochondrial transcription termination and primer formation. *Proc. Natl. Acad. Sci. USA* **107**, 16072–16077 (2010). 20798345 10.1073/pnas.1006026107 PMC2941323

[21] Dong, D. W. et al. Association of G-quadruplex forming sequences with human mtDNA deletion breakpoints. *BMC Genomics* **15**, 677 (2014). 25124333 10.1186/1471-2164-15-677 PMC4153896

[22] Vögtle, F. N. et al. Landscape of submitochondrial protein distribution. *Nat. Commun*. **8**10.1038/s41467-017-00359-0 (2017). 10.1038/s41467-017-00359-0 PMC5561175 28819139

[23] Rath, S. et al. MitoCarta3.0: an updated mitochondrial proteome now with sub-organelle localization and pathway annotations. *Nucleic Acids Res.* **49**, D1541–D1547 (2021). 33174596 10.1093/nar/gkaa1011 PMC7778944

[24] Bulthuis, E. P. et al. Stress‐dependent macromolecular crowding in the mitochondrial matrix. *EMBO J.* **42**10.15252/embj.2021108533 (2023). 10.15252/embj.2021108533 PMC10068333 36825437

[25] Tinoco, I. Jr., Uhlenbeck, O. C. & Levine, M. D. Estimation of secondary structure in ribonucleic acids. *Nature* **230**, 362–367 (1971). 4927725 10.1038/230362a0

[26] Tinoco, I. et al. Improved estimation of secondary structure in ribonucleic acids. *Nat. New Biol.* **246**, 40–41 (1973). 4519026 10.1038/newbio246040a0

[27] Breslauer, K. J., Frank, R., Blöcker, H. & Marky, L. A. Predicting DNA duplex stability from the base sequence. *Proc. Natl. Acad. Sci. USA* **83**, 3746–3750 (1986). 3459152 10.1073/pnas.83.11.3746 PMC323600

[28] Freier, S. M. et al. Improved free-energy parameters for predictions of RNA duplex stability. *Proc. Natl. Acad. Sci. USA* **83**, 9373–9377 (1986). 2432595 10.1073/pnas.83.24.9373 PMC387140

[29] Sugimoto, N. et al. Thermodynamic parameters to predict stability of RNA/DNA hybrid duplexes. *Biochemistry* **34**, 11211–11216 (1995). 7545436 10.1021/bi00035a029

[30] Banerjee, D. et al. Improved nearest-neighbor parameters for the stability of RNA/DNA hybrids under a physiological condition. *Nucleic Acids Res.* **48**, 12042–12054 (2020). 32663294 10.1093/nar/gkaa572 PMC7708073

[31] Ghosh, S. et al. Nearest-neighbor parameters for the prediction of RNA duplex stability in diverse in vitro and cellular-like crowding conditions. *Nucleic Acids Res.* **51**, 4101–4111 (2023). 36718808 10.1093/nar/gkad020 PMC10201447

[32] Ghosh, S. et al. Nearest-neighbor parameters for predicting DNA duplex stability in diverse molecular crowding conditions. *Proc. Natl. Acad. Sci. USA* **117**, 14194–14201 (2020). 32522884 10.1073/pnas.1920886117 PMC7321957

[33] Ghosh, S., Takahashi, S., Ohyama, T., Liu, L. & Sugimoto, N. Elucidating the role of groove hydration on stability and functions of biased DNA duplexes in cell-like chemical environments. *J. Am. Chem. Soc.* **146**, 32479–32497 (2024). 39505325 10.1021/jacs.4c09388 PMC11613987

[34] Gonzalez-Tello, P., Camacho, F. & Blazquez, G. Density and viscosity of concentrated aqueous solutions of polyethylene glycol. *J. Chem. Eng. Data* **39**, 611–614 (1994).

[35] Nakano, S.-I., Karimata, H., Ohmichi, T., Kawakami, J. & Sugimoto, N. The effect of molecular crowding with nucleotide length and cosolute structure on DNA duplex stability. *J. Am. Chem. Soc.* **126**, 14330–14331 (2004). 15521733 10.1021/ja0463029

[36] Ghosh, S. et al. Validation of the nearest-neighbor model for Watson-Crick self-complementary DNA duplexes in molecular crowding condition. *Nucleic Acids Res.* **47**, 3284–3294 (2019). 30753582 10.1093/nar/gkz071 PMC6468326

[37] Trajkovski, M. et al. Pursuing origins of (poly)ethylene glycol-induced G-quadruplex structural modulations. *Nucleic Acids Res.* **46**, 4301–4315 (2018). 29648656 10.1093/nar/gky250 PMC5934638

[38] Fushimi, K. & Verkman, A. S. Low viscosity in the aqueous domain of cell cytoplasm measured by picosecond polarization microfluorimetry. *J. Cell Biol.* **112**, 719–725 (1991). 1993739 10.1083/jcb.112.4.719 PMC2288848

[39] Hertzog, M. & Erdel, F. The material properties of the cell nucleus: A matter of scale. *Cells* **12**, 1958 (2023). 37566037 10.3390/cells12151958 PMC10416959

[40] Tateishi-Karimata, H., Kawauchi, K. & Sugimoto, N. Destabilization of DNA G-Quadruplexes by chemical environment changes during tumor progression facilitates transcription. *J. Am. Chem. Soc.* **140**, 642–651 (2018). 29286249 10.1021/jacs.7b09449

[41] Bharti, S. K. et al. DNA sequences proximal to human mitochondrial DNA deletion breakpoints prevalent in human disease form G-quadruplexes, a class of DNA structures inefficiently unwound by the mitochondrial replicative twinkle helicase. *J. Biol. Chem.* **289**, 29975–29993 (2014). 25193669 10.1074/jbc.M114.567073 PMC4208006

[42] Yett, A., Lin, L. Y., Beseiso, D., Miao, J. & Yatsunyk, L. A. N-methyl mesoporphyrin IX as a highly selective light-up probe for G-quadruplex DNA. *J. Porphyr. Phthalocyanines* **23**, 1195–1215 (2019). 34385812 10.1142/s1088424619300179 PMC8356643

[43] Kreig, A. et al. G-quadruplex formation in double strand DNA probed by NMM and CV fluorescence. *Nucleic Acids Res.* **43**, 7961–7970 (2015). 26202971 10.1093/nar/gkv749 PMC4652765

[44] Del Villar-Guerra, R., Trent, J. O. & Chaires, J. B. G-Quadruplex secondary structure obtained from circular dichroism spectroscopy. *Angew. Chem. Int. Ed.* **57**, 7171–7175 (2018). 10.1002/anie.201709184 PMC5920796 29076232

[45] Yoshinaga, N. et al. Design of an artificial peptide inspired by transmembrane mitochondrial protein for escorting exogenous DNA into the mitochondria to restore their functions by simultaneous multiple gene expression. *Adv. Funct. Mater.* **34**, 2306070 (2024).

[46] Yoshinaga, N. & Numata, K. Rational designs at the forefront of mitochondria-targeted gene delivery: recent progress and future perspectives. *ACS Biomater. Sci. Eng.* **8**, 348–359 (2022). 34979085 10.1021/acsbiomaterials.1c01114

[47] Gray, D. M., Hung, S. H. & Johnson, K. H. Absorption and circular dichroism spectroscopy of nucleic acid duplexes and triplexes. *Methods Enzymol.* **246**, 19–34 (1995). 10.1016/0076-6879(95)46005-5 7538624

[48] Huguet, J. M. et al. Single-molecule derivation of salt dependent base-pair free energies in DNA. *Proc. Natl. Acad. Sci. USA* **107**, 15431–15436 (2010). 20716688 10.1073/pnas.1001454107 PMC2932562

[49] Chuah, J.-A., Matsugami, A., Hayashi, F. & Numata, K. Self-assembled peptide-based system for mitochondrial-targeted gene delivery: functional and structural insights. *Biomacromol.* **17**, 3547–3557 (2016). 10.1021/acs.biomac.6b01056 27696822

