# Supplementary material for: Predictability of environment-dependent formation of G-quadruplex DNAs in human mitochondria
Source: Commun Chem. 2025 May 3;8:135. doi: 10.1038/s42004-025-01532-z (PMC12049430; doi:10.1038/s42004-025-01532-z)
Supplement: Supplementary file 1 — Supplementary information [file 42004_2025_1532_MOESM1_ESM.pdf]

Supplementary Information

Predictability of environment-dependent formation of G-quadruplex DNAs in human mitochondria

Lutan Liu,[1,#] Shuntaro Takahashi,[1,2,#,*] Sarptarshi Ghosh,[1] Tamaki Endoh,[1,2] Naoto Yoshinaga,[3,4] Keiji Numata[3,4,5] and Naoki Sugimoto[1*]

[1]FIBER (Frontier Institute for Biomolecular Engineering Research), Konan University, 7-1-20 Minatojima-Minamimachi, Chuo-ku, Kobe 650-0047, Japan

[2]FIRST (Graduate School of Frontiers of Innovative Research in Science and Technology), Konan University, 7-1-20 Minatojima-Minamimachi, Chuo-ku, Kobe 650-0047, Japan

[3]Biomacromolecule Research Team, RIKEN Center for Sustainable Resource Science, Wako-shi, Saitama 351-0198, Japan.

[4]Institute for Advanced Biosciences, Keio University, Tsuruoka-shi, Yamagata 997-0017, Japan.

[5]Department of Material Chemistry, Kyoto University, Kyoto-shi, Kyoto 606-8501, Japan.

[#] *These authors contributed equally.*

* *These authors jointly supervised this work.*

Email: shtakaha@konan-u.ac.jp, sugimoto@konan-u.ac.jp

Table S1. Thermodynamic parameters measured for DNA duplex formation in the absence and presence of 40% PEG200 and 20 wt% PEG8000 in 10 mM phosphate buffer (pH 7.0) with 100 mM NaCl and 1 mM EDTA.

Sequences[a]	Measured in non-crowding condition[b]			Measured in the presence of 40 wt% PEG200[b]			Measured in the presence of 20 wt% PEG8000		
	$\Delta H°$ (kcal mol^{-1})	$T\Delta S°$ (kcal mol^{-1})	$\Delta G°_{37}$ (kcal mol^{-1})	$\Delta H°$ (kcal mol^{-1})	$T\Delta S°$ (kcal mol^{-1})	$\Delta G°_{37}$ (kcal mol^{-1})	$\Delta H°$ (kcal mol^{-1})	$T\Delta S°$ (kcal mol^{-1})	$\Delta G°_{37}$ (kcal mol^{-1})
Unbiased d(ATGAGCTCAT)	-68.6 ± 3.1	-7.7 ± 0.3	-7.7 ± 0.3	-71.8 ± 0.9	-66.8 ± 0.9	-5.0 ± 0.1	-85.1 ± 4.1	-75.6 ± 3.7	-9.4 ± 0.4
GC-biased d(GCGCCGC)	-58.4 ± 2.5	-48.8 ± 2.1	-9.6 ± 0.5	-61.1 ± 2.1	-52.9 ± 1.9	-8.2 ± 0.6	-54.3 ± 2.9	-43.9 ± 2.3	-10.4 ± 0.6

[a]The DNA duplex consists of a DNA strand and its complementary DNA strand

[b]The thermodynamic parameters were reported in the previous study[1].

Table S2. Viscosity of the buffer with the addition of respective crowding agents at 37°C

Buffer[a]	Viscosity (cP)
30 wt% 1,3-PDO	1.81
45 wt% 1,3-PDO	2.52
60 wt% 1,3-PDO	4.01
30 wt% 1,3-PDO + 0.5 wt% PEG8000	3.61
30 wt% 1,3-PDO + 1 wt% PEG8000	2.37

[a] Contains 10 mM Na_2HPO_4, 1 mM EDTA, and 150 mM KCl

Table S3. DNA sequences used in the NMM and CD assays

Name[a]	Sequence (5'–3')
L-s	TTGGGTTGTAACTATCGAGG
L-as	CCTCGATAGTTACAACCCAA
D1-s	GGGAGCCAGGGACGGCCGGG
D1-as	CCCGGCCGTCCCTGGCTCCC
D2-s	GGGGACAGGGGCGGGGTGGG
D2-as	CCCACCCCGCCCCTGTCCCC
D4-s	GGGGGGATCAGCGGGAGGGCTGGG
D4-as	CCCAGCCCTCCCGCTGATCCCCCC
mt6363-s	GGGACGCGGGCGGGGGGATATAGGG
mt6363-as	CCCTATATCCCCCCGCCCGCGTCCC
CSBII-s	GCGGGGGAGGGGGGGTTTG
CSBII-as	CAAACCCCCCCTCCCCCGC
KSS-s	GGGGAGGGGTGTTTAAGGGGTGGCTAGGG
KSS-as	CCCTAGCCAACCCCTTAAACACCCCTCCCC
PMPS-s	GGGACGCGGGCGGGGATATAGGG
PMPS-as	CCCTATATCCCCGCCCGCGTCCC
HRCC-s	GGGGGTTGGGTATGGGGAGGGGGG
HRCC-as	CCCCCCTCCCCAACCCAACCCCC

[a] Sequences indicated by "-s" are complementary to those indicated by "-as".

Table S4. Average % intensity of the ss region within the dsDNA lane of the native PAGE analysis

Sequence	% intensity with respect to the ds DNA band	% intensity with respect to the G-rich ss DNA band
L	-0.6 ± (1.4)	-2.6 ± (5.4)
D1	2.3 ± (1.5)	4.8 ± (3.0)
D2	-0.4 ± (0.4)	-0.8 ± (0.5)
mt6363	2.9 ± (0.2)	5.6 ± (0.6)
PMPS	6.2 ± (2.7)	9.9 ± (5.4)
D4	0.8 ± (0.7)	1.5 ± (1.2)
KSS	-0.6 ± (0.2)	-1.1 ± (0.2)
HRCC	-0.2 ± (0.3)	-0.4 ± (0.8)
CSBII	-0.1 ± (0.2)	-0.1 ± (0.3)

*SD is indicated in parentheses

Table S5. Relative Increase in G4 formation in mitochondria-like conditions with respect to nucleus-like condition in the presence of 20% excess C-rich strand

Solution	D2	D4	mt6363	CSBII
30 wt% 1,3-PDO (nucleus-like condition)	1	1	1	1
45 wt% 1,3-PDO	1.01	2.80	2.20	0.97
60 wt% 1,3-PDO	1.15	3.29	3.43	1.61
30 wt% 1,3-PDO + 0.5 wt% PEG8000	0.99	0.75	1.11	1.12
30 wt% 1,3-PDO + 1 wt% PEG8000	0.85	1.05	1.06	1.02

Table S6. Sequences of DNA primer used for construction of plasmid DNAs to express GFP in mitochondria

Name	Sequence (5'–3') [a]
2REGFPfwd	CAAAGCTTAGCTGAGGTCTCATATGGTGAGCAAGGGC GAGG
pDONRtermrev	CCAGAGCTGCAGCTGGATGG
2REGFPfwd-s	CTGAGGTCTCATATGGTG
pDONRpromfwd	CGTTAACGCTAGCATGGATCTCGG
pDONR-cox22RErev-s	CATATGAGACCTCAGCTAAGCTTTGTTAATTG
pDONRtermrev-f	CAGCTGCAGCTCTGGCCC
pDONRpromfwd-r	ATGCTAGCGTTAACGCGAGAGTAG
pDONR-cox2gfpfwd	TAAAACCCAGCTTTCTTGTACAAAGTGGG
pDONR-cox2gfp-15rev	GCCCTTGCTCACCATTGTTAATTGTAATCTTAATAAAT CTAACATTTTAATAAATCTTAACC
InFGFPfwd	ATGGTGAGCAAGGGCGAGG
cox2GFPInFrev	GAAAGCTGGGTTTTACTTGTACAGCTCGTCCAT
L-s	[PHO]AGCTTGCCGTTTCGTAGTAATTGGGTTGTAACTA TCGAGGCA
L-as	[PHO]TATGCCTCGATAGTTACAACCCAATTACTACGAA ACGGCA
D1-s	[PHO]AGCTTGCCGTTTCGTAGTAAGGGAGCCAGGGA CGGCCGGGCA
D1-as	[PHO]TATGCCCGGCCGTCCCTGGCTCCCTTACTACGA AACGGCA
D2-s	[PHO]AGCTTGCCGTTTCGTAGTAAGGGGACAGGGGC GGGGTGGGCA
D2-as	[PHO]TATGCCCACCCCGCCCCTGTCCCCTTACTACGA AACGGCA
D4-s	[PHO]AGCTTGCCGTTTCGTAGGGGGGATCAGCGGGA GGGCTGGGCA
D4-as	[PHO]TATGCCCAGCCCTCCCGCTGATCCCCCCTACGA AACGGCA

[a][PHO] indicates the phosphate modification.

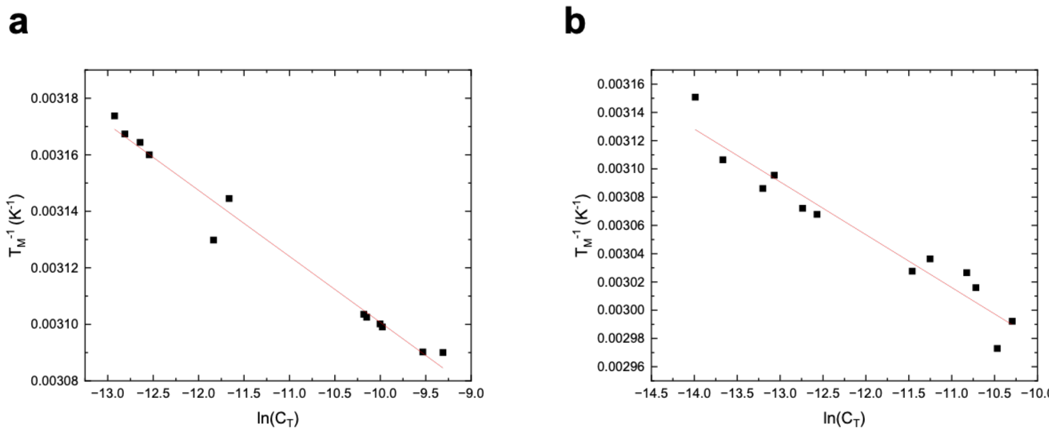

Fig. S1. T_m^{-1} versus ln (C_t) plots of unbiased and biased duplex DNA. T_m^{-1} versus ln (C_t) plots. T_m^{-1} versus ln (C_t) plots of the unbiased sequence d(ATGAGCTCAT) and the biased sequence d(GCGCCGC) in the presence of 20 wt% PEG8000.

a

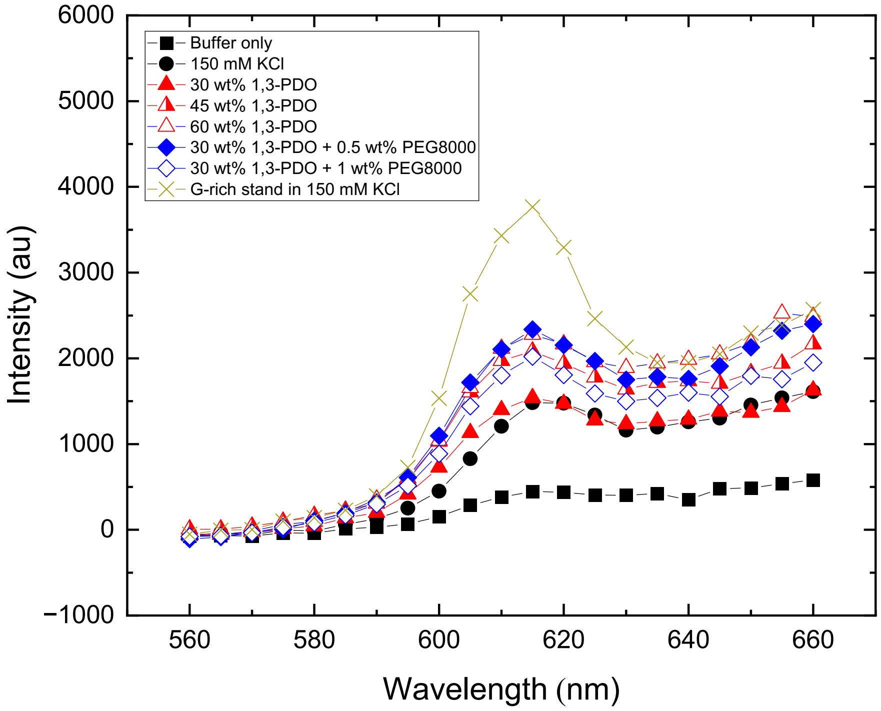
Buffer only
150 mM KCl
30 wt% 1,3-PDO
45 wt% 1,3-PDO
60 wt% 1,3-PDO
30 wt% 1,3-PDO + 0.5 wt% PEG8000
30 wt% 1,3-PDO + 1 wt% PEG8000
G-rich stand in 150 mM KCl
Intensity (au)
Wavelength (nm)
6000
5000
4000
3000
2000
1000
0
−1000
560
580
600
620
640
660

Continued

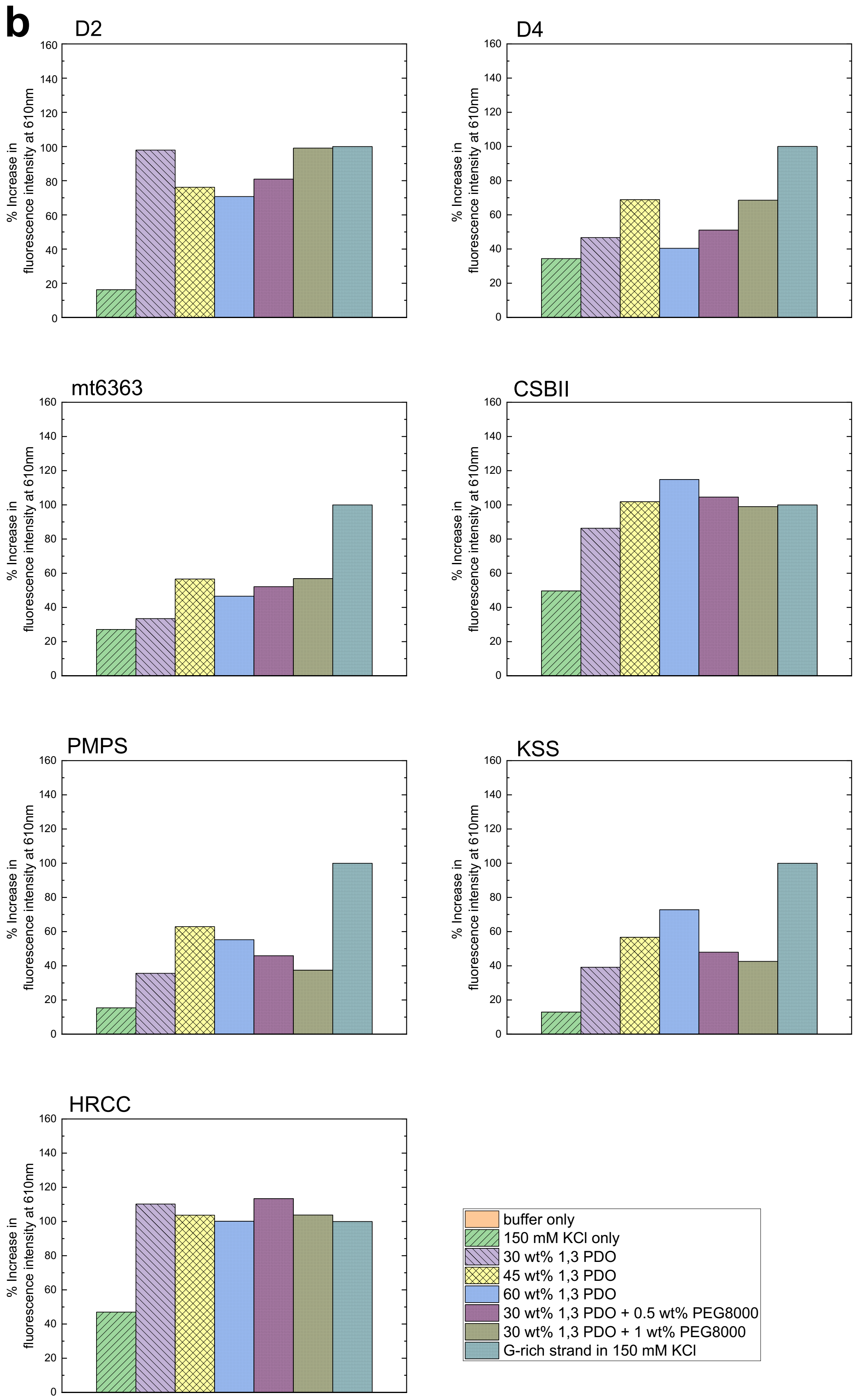
b
D2
D4
mt6363
CSBII
PMPS
KSS
HRCC
% Increase in fluorescence intensity at 610nm
0
20
40
60
80
100
120
140
160
buffer only
150 mM KCl only
30 wt% 1,3 PDO
45 wt% 1,3 PDO
60 wt% 1,3 PDO
30 wt% 1,3 PDO + 0.5 wt% PEG8000
30 wt% 1,3 PDO + 1 wt% PEG8000
G-rich strand in 150 mM KCl

Fig. S2. NMM assay and analysis of G4 formation by double-stranded DNA in mitochondria-mimicking conditions. (**a**) Representative fluorescence spectra of NMM-treated double-stranded mt6363 sequences. The spectra were measured in 10 mM sodium phosphate and 1 mM EDTA buffer (pH 7.0) with the following additions: (i) buffer only (black square), (ii) 150 mM KCl (black circle), (iii) 150 mM KCl and 30 wt% 1,3-PDO (red closed triangle), (iv) 150 mM KCl and 45 wt% 1,3-PDO (red striped triangle), (v) 150 mM KCl and 60 wt% 1,3-PDO (red open triangle), (vii) 150 mM KCl, 30 wt% 1,3-PDO and 0.5 wt% PEG8000 (blue closed diamond), and (viii) 150 mM KCl, 30 wt% 1,3-PDO and 1 wt% PEG8000. Additionally, the spectra of single-stranded -s strands in the buffer with 150 mM KCl without cosolutes are also shown (shown as "X"). (**b**) The relative NMM fluorescence intensity at 610 nm of the D2, D4, mt6363, CSBII, KSS, PMPS, and HRCC sequences. The signals of the duplex in the absence of KCl and cosolutes are defined as 0%, and the signals of the single-stranded G-rich strand in the presence of 150 mM KCl are defined as 100%.

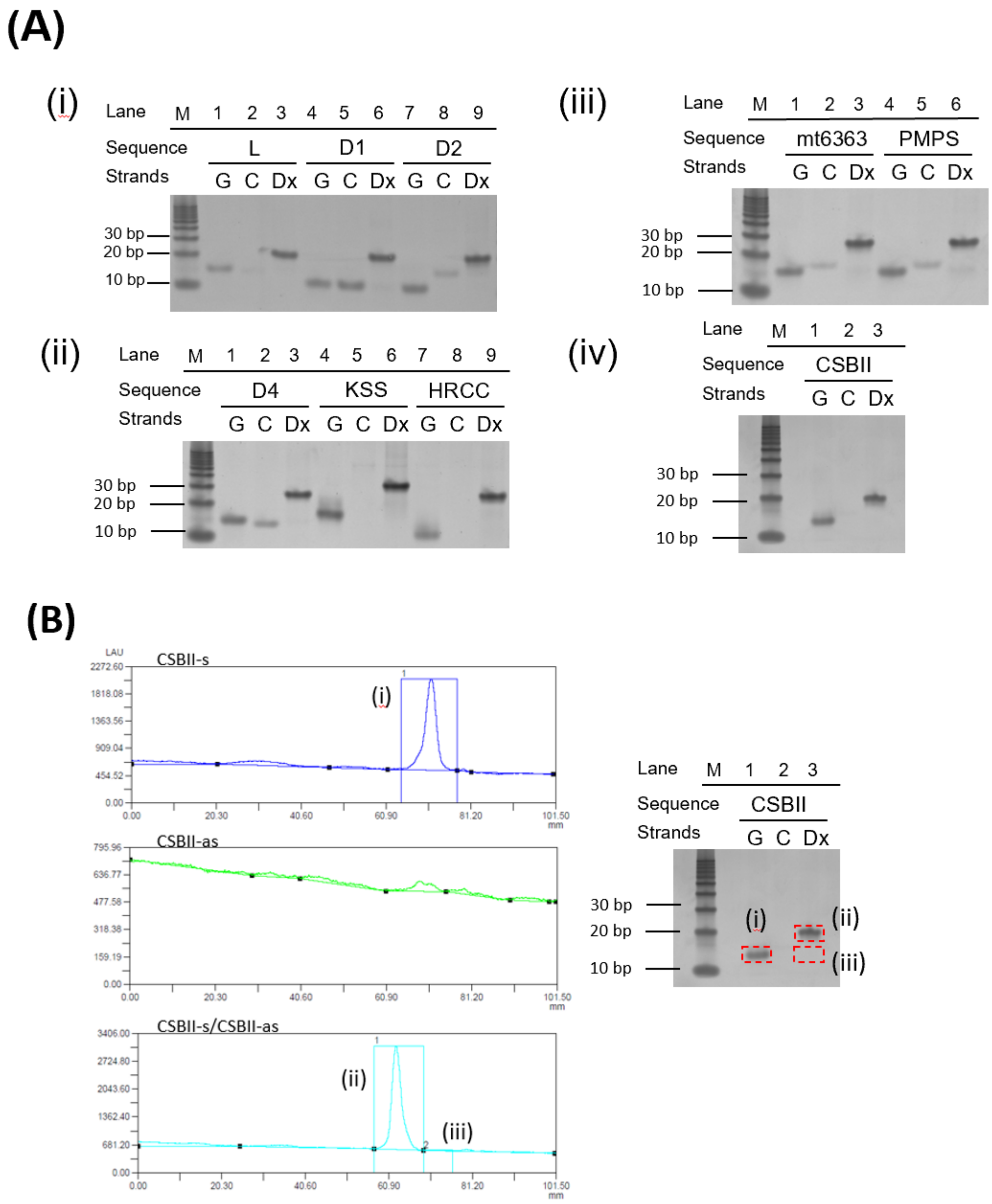

Fig. S3. Native PAGE analysis of double-stranded DNA with G4 forming sequences. **(A)** Representative native PAGE analysis of (i) L-s (lane 1), L-as (lane 2), L-s/L-as dsDNA (lane 3), D1-s (lane 4), D1-as (lane 5), D1-s/D1-as dsDNA (lane 6), D2-s (lane 7), D2-as (lane 8), and D2-s/D2-as dsDNA (lane 9); (ii) D4-s (lane 1), D4-as (lane 2), D4-s/D4-as dsDNA (lane 3), KSS-s (lane 4), KSS-as (lane 5), KSS-s/KSS-as dsDNA (lane 6), HRCC-s (lane7), HRCC-as (lane 8), and HRCC-s/HRCC-as dsDNA (lane 9); (iii) mt6363-s (lane1), mt6363-as

(lane 2), mt6363-s/mt6363-as dsDNA (lane 3), PMPS-s (lane 4), PMPS-as (lane 5), PMPS-s/PMPS-as dsDNA (lane 6); (iv) CSBII-s (lane 1), CSBII-as (lane 2), and CSBII-s/CSB-as dsDNA (lane 3). In the lane labels, the G-rich -s strands are denoted as G, C-rich -as strands are denoted as C, and the dsDNA are denoted as Dx. M represents the DNA base pair (bp) marker. The solutions were prepared in 10mM Li_2HPO_4, 1mM Li_2EDTA, and 100mM LiCl at pH 7.0. Gel electrophoresis was performed in triplicate in 15% polyacrylamide gel at 4 ℃ in TBE buffer. For ssDNA, 2 μL of 0.25 μM DNA was loaded, and for dsDNA, 2 μL of 0.50 μM DNA was loaded. **(B)** Representative peak profiles of lanes 1-3, where peaks (i), (ii) and (iii) represents CSBII-s band lane 1, CSBII-ds band in lane 3, and CSBII-s band in lane 3, respectively. The x-axis is the distance of the lane in mm. y-axis is LAU (Linear arbitrary unit) that represents region intensity of the lane. Peak intensities were computed as the area under the peak.

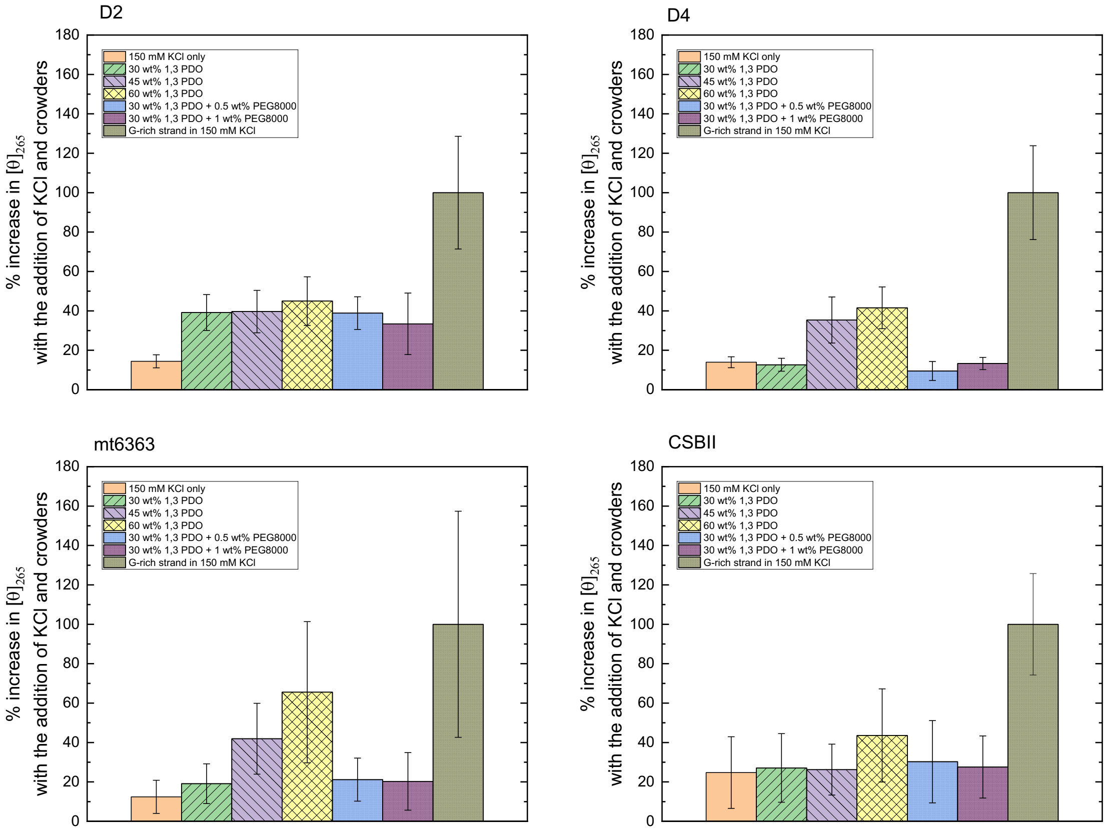

Fig. S4. CD analysis of G4 formation by double-stranded DNA with excess C-rich DNA in mitochodnria-mimicking conditions. The relative increase in the molar ellipticity at 265 nm of the double-stranded D2, D4, mt6363, and CSBII sequences with 20% excess C-rich strand in 10 mM Na_2HPO_4 and 1 mM EDTA buffer (pH 7.0) with the additions of the following: 150 mM KCl (orange), 150 mM KCl and 30 wt% 1,3-PDO (green), 150 mM KCl and 45% 1,3-PDO (purple), 150 mM KCl and 60 wt% 1,3-PDO (yellow), 150 mM KCl, 30 wt% 1,3-PDO, and 0.5 wt% PEG8000 (blue), and 150 mM KCl, 30 wt% 1,3-PDO, and 1 wt% PEG8000 (pink). D2-s, D4-s, mt6363-s, and CSBII-s strands in the buffer and 150 mM KCl without crowders are also shown (Olive). For both cases, the signals of duplexes in the absence of KCl and cosolutes are defined as 0%, while the signals of single-stranded G-rich strands in the presence of 150 mM KCl are defined as 100%. The experiment was conducted in triplicate, and the standard deviations are represented by the error bars.

Supplementary References

1 Ghosh, S., Takahashi, S., Ohyama, T., Liu, L. & Sugimoto, N. Elucidating the Role of Groove Hydration on Stability and Functions of Biased DNA Duplexes in Cell-Like Chemical Environments. *J. Am. Chem. Soc.* **146**, 32479-32497 (2024). https://doi.org/10.1021/jacs.4c09388
