# Supplementary material for: Predictability of environment-dependent formation of G-quadruplex DNAs in human mitochondria
Source: Commun Chem. 2025 May 3;8:135. doi: 10.1038/s42004-025-01532-z (PMC12049430; doi:10.1038/s42004-025-01532-z)
Supplement: Supplementary file 2 — Description of Additional Supplementary Files [file 42004_2025_1532_MOESM2_ESM.pdf]

**Description of Additional Supplementary Files**

File name- Supplementary Data 1

File description- Source data - CD spectra

File name- Supplementary Data 2

File description- Source data - GFP reporter assay

File name- Supplementary Data 3

File description- Source data - Native PAGE

File name- Supplementary Data 4

File description- Source data - NMM assay
